# Drivers of downloading and reasons for not downloading COVID-19 contact tracing and exposure notification apps: A national cross-sectional survey

**DOI:** 10.1371/journal.pone.0269783

**Published:** 2022-07-15

**Authors:** Golden Gao, Raynell Lang, Robert J. Oxoby, Mehdi Mourali, Hasan Sheikh, Madison M. Fullerton, Theresa Tang, Braden J. Manns, Deborah A. Marshall, Jia Hu, Jamie L. Benham

**Affiliations:** 1 Department of Family Medicine, Faculty of Health Sciences, Queen’s University, Kingston, ON, Canada; 2 Department of Medicine, Cumming School of Medicine, University of Calgary, Calgary, AB, Canada; 3 Department of Economics, Faculty of Arts, University of Calgary, Calgary, AB, Canada; 4 Haskayne School of Business, University of Calgary, Calgary, AB, Canada; 5 Department of Family and Community Medicine, University of Toronto, Toronto, ON, Canada; 6 Department of Community Health Sciences, Cumming School of Medicine, University of Calgary, Calgary, AB, Canada; University of Montreal, CANADA

## Abstract

**Background:**

Bluetooth-enabled smartphone apps have been developed and implemented in different sites globally to help overcome capacity limitations of traditional interview-based COVID-19 contact tracing. Two apps are currently available in Canada: ABTraceTogether exclusively in Alberta and COVID Alert in nine other provinces and territories. This study aims to examine factors associated with downloading of these apps to inform targeted promotion and marketing to increase app uptake.

**Methods:**

We performed a cross-sectional survey with adult participants (≥18 years old) from an online national panel. Participants were asked if they had downloaded an app and, if applicable, reasons for not downloading. Logistic regression was used to identify sociodemographic factors and trusted information sources associated with downloading and reasons for not downloading.

**Results:**

Of the included 4,503 respondents (36% response rate), 1,394 (31%) had downloaded an app. Demographic and socioeconomic factors positively associated with app download were: 1) being female, 2) higher household income, 3) higher education level attained, and 4) more liberal political views. The odds of downloading an app were higher for participants who trusted health-related information sources, and lower for those who trusted internet searches, family and friend, or Facebook. The most cited reasons for not downloading were related to data security concerns and perceived lack of benefit from the apps.

**Interpretation:**

These findings identify sociodemographic segments with the lowest app uptake, reasons for not downloading and trusted information sources to inform targeted promotion and marketing strategies to improve uptake of apps to facilitate contact tracing.

## Introduction

A core public health measure for slowing transmission of COVID-19 is contact tracing, which involves interviewing persons with COVID-19 to identify individuals who they have been in contact with and providing guidance for symptom monitoring, testing, and/or quarantining [1]. Contact tracing is effective in slowing COVID-19 transmission if done in a timely and comprehensive fashion in conjunction with quarantining, as demonstrated in South Korea, Vietnam, Japan, and Taiwan [2].

Traditional contact tracing is resource-intensive and limited by organizational capacity, contact tracer experience, process delays, recall errors, and missing contacts for exposures in public places [2, 3]. One solution is using digital contact tracing or exposure notification apps that can detect distances and timing of contact between two smartphones using Bluetooth technology [4]. Early simulation models of COVID-19 transmission show that ~60% population uptake of these apps may slow regional transmission [5]. More recent models suggest that lower levels of adoption could still be effective, especially when combined with adequate testing capacity and population-level interventions [6–8].

Canada has developed two apps including COVID Alert [9] for use in eight provinces and the Northwest Territories, and ABTraceTogether [10] for use in the province of Alberta. Released in July 2020, COVID Alert uses decentralized Bluetooth technology developed by Google/Apple [11, 12]. ABTraceTogether, released in May 2020, uses decentralized Bluetooth technology developed by the Government of Alberta [10]. As of February 2022, >6.8 million Canadians (18% population uptake) had downloaded COVID Alert [9] compared with ~317,000 downloads (7% population uptake) for ABTraceTogether [13].

Understanding public perception of these apps and reasons for downloading or not downloading them is important for tailoring app design and promotion to increase uptake and effectiveness in slowing COVID-19 transmission. Contact tracing applications have been used for other infectious diseases with variable success [14, 15]. Prior to wide app implementation, studies from Europe and North America showed mixed public perception with issues of trust (i.e., security and privacy concerns, fear of surveillance) being common reasons for individuals not adopting them [16–20]. Evidence from European countries has emerged to describe characteristics of app adopters and drivers of uptake following expanded implementation throughout the second half of 2020 [21, 22]. A study evaluating nine different European national apps for COVID-19 contact tracing revealed users were generally dissatisfied, expressing concerns about usability and effectiveness of the apps [23].

While small-scale Canadian studies have been done to evaluate design factors that influence user perception and adoption of contact tracing apps, to our knowledge, there have been no large-scale studies that characterize Canadians who have and have not downloaded these apps and the reasons why [24, 25]. We aim to examine sociodemographic factors associated with Canadians who download these apps and investigate barriers and facilitators to app download.

## Methods

### Study design

In the summer and fall of 2020, the governments of Canada and Alberta were actively encouraging Canadians and Albertans to download and use COVID Alert and ABTraceTogether to reduce the transmission of COVID-19 [26, 27] as Canada entered the second wave of the COVID-19 pandemic and case counts were starting to rise across the country [28]. At this time, non-pharmacologic interventions including contact tracing and exposure notification apps were the primary means of preventing COVID-19 transmission as COVID-19 vaccines were in development but had not yet been approved for use in Canada [29]. In this context, we designed an online, cross-sectional survey to assess reasons for downloading and not downloading COVID Alert and ABTraceTogether. Study questions were determined based on a review of the current literature as well as prior focus groups and a pilot survey that were completed in Alberta, Canada in the summer of 2020 [30, 31]. The survey questions have previously been published [32].

The University of Calgary Conjoint Health Research Ethics Board approved this study (REB20-1228). Informed consent was obtained and participation was voluntary. We used the Strengthening the Reporting of Observational Studies in Epidemiology (STROBE) checklist to report our findings [33].

### Survey administration and participants

The survey was programmed via Askia and administered through Platform One by the Angus Reid Institute [34–36], a national, not-for-profit, research foundation, from October 27 to November 2, 2020 during the second wave of the COVID-19 pandemic in Canada [28]. It was administered in English and French. Survey invitations were sent to 14,887 potential participants to obtain a sample size of 4500 participants. These potential participants were randomly selected from the Angus Reid Forum, a forum of 70,000 individuals from all major demographic groups to ensure a representative sample of the Canadian population [37]. Sampling was stratified to get equal numbers of Alberta residents and residents of the other Canadian provinces to allow for evaluation of both ABTraceTogether (only available in Alberta), and COVID Alert (available in eight provinces and the Northwest Territories). To be eligible, participants were required to be aged ≥18 years, live in a Canadian province, speak English or French, and have internet access. Participants were remunerated for their participation consistent with Angus Reid Forum policy [34].

### Measurement

The main outcome measure was whether participants had downloaded an app, a proxy for app adoption. Participants were asked if they had downloaded ABTraceTogether (in Alberta) or COVID Alert (outside of Alberta) at any point. Individuals who had not downloaded an app were asked their reasons for not downloading under four themes (i.e., data security, perceived lack of benefit, digital barriers and lack of awareness), and if they planned to download an app in the future. All participants were asked their trusted sources of COVID-19 information. Sociodemographic factors were collected based on a literature search and previous focus groups [30] for factors that may be associated with downloading a contact tracing app. These factors included sex, age, province of residence, household income, highest level of education, ethnicity, and political leaning.

### Statistical analysis

Descriptive statistics (percentage frequencies) were calculated for all demographic characteristics and reasons for not downloading an app. Persons who had downloaded an app were compared to persons who had not. To inform targeted messaging designed to increase app download, we estimated the association (odds ratio, or OR) using logistic regression between demographic factors and 1) downloading an app, 2) reasons for not downloading an app, and 3) consideration of downloading an app in the future. Estimates of association between downloading an app and demographic factors were adjusted for all demographic factors and reported as an adjusted OR (aOR). Covariates included in these adjusted models were chosen *a priori* based on literature review, prior focus groups and a pilot survey [31, 38]. To inform messaging content and delivery platforms, logistic regression was used to explore the association between 1) consideration of downloading an app in the future and reasons for not downloading, and 2) trusted sources of COVID-19 information and downloading an app. These estimates were adjusted for sex, province, education level, political leaning and income level based on a backward stepwise regression model. Data on political leaning were missing for five participants. Analyses were conducted using STATA Version 15.1 (StataCorp, College Station, TX, USA). A *P*-value of <0.05 was set as significant. Each OR and aOR was reported with the associated 95% confidence interval (95%CI).

## Results

The survey study was distributed to 14,887 participants and had 5,359 respondents (36% response rate) with 1,388 (26% of respondents) excluded due to incomplete survey responses. Overall, 4,503 participants completed the survey and were included in the analysis (Table 1). There was an even distribution of male (49%) and female (51%) participants with median age of 46 years old (IQR 33–61). Most of the participants received some post-secondary education (80%) and identified as Caucasian (85%).

**Table 1:** Participant characteristics associated with downloading apps.

Characteristic	Total N = 4503 (%)	Downloaded a Contact Tracing App		Crude OR*	95% CI	Adjusted OR‡	95% CI
		Yes (N = 1394)	No (N = 3109)				
Biologic Sex, N (%)							
Female (REF)	2298 (51)	772 (34)	1526 (66)	1.00		1.00	
Male	2205 (49)	622 (28)	1583 (72)	0.77	0.68–0.88	0.86	0.75–0.99
Age (years), N (%)							
18–34 (REF)	1341 (30)	467 (35)	874 (65)	1.00		1.00	
35–54	1589 (35)	501 (32)	1088 (68)	0.86	0.74–1.01	1.04	0.87–1.23
55+	1573 (35)	426 (27)	147 (73)	0.70	0.59–0.81	0.94	0.78–1.12
Province, N (%)							
Alberta^^^ (REF)	2003 (44)	452 (23)	1551 (77)	1.00		1.00	
British Columbia^^^^	502 (11)	102 (20)	400 (80)	0.88	0.69–1.11	0.74	0.57–0.96
Prairie Provinces^+^	445 (10)	160 (36)	285 (64)	1.93	1.55–2.40	2.33	1.84–2.95
Ontario	800 (18)	389 (49)	411 (51)	1.89	1.43–2.49	3.20	2.65–3.86
Quebec	502 (11)	202 (40)	300 (60)	3.25	2.73–3.86	2.26	1.81–2.83
Atlantic Provinces^+^	251 (6)	89 (36)	162 (64)	2.31	1.88–2.84	1.87	1.39–2.52
**Household Income (CAD $), N (%)**							
**<$50,000**	**951 (21)**	**248 (26)**	**703 (74)**	**1.00**		**1.00**	
**$50,000-$99,999**	**1332 (30)**	**428 (32)**	**904 (68)**	**1.34**	**1.12–1.61**	**1.42**	**1.16–1.74**
**$100,000-$199,999**	**1354 (30)**	**486 (36)**	**868 (64)**	**1.59**	**1.32–1.90**	**1.70**	**1.39–2.10**
**≥$200,000**	**214 (5)**	**78 (36)**	**136 (64)**	**1.63**	**1.19–2.22**	**2.06**	**1.44–2.95**
**Rather Not Say**	**652 (15)**	**154 (24)**	**498 (76)**	**0.88**	**0.70–1.10**	**1.11**	**0.86–1.43**
**Highest Level of Education, N (%)**							
**High School Graduate or less**	**898 (20)**	**186 (21)**	**712 (79)**	**1.00**		**1.00**	
**Some College or Trade School**	**840 (19)**	**208 (25)**	**632 (75)**	**1.26**	**1.01–1.58**	**1.18**	**0.93–1.51**
**College or Trade School**	**997 (22)**	**298 (30)**	**699 (70)**	**1.63**	**1.32–2.01**	**1.48**	**1.18–1.86**
**Some University**	**454 (10)**	**163 (36)**	**291 (64)**	**2.14**	**1.67–2.75**	**1.71**	**1.31–2.25**
**University Degree**	**1314 (29)**	**539 (41)**	**775 (59)**	**2.66**	**2.19–3.24**	**1.84**	**1.47–2.29**
**Ethnicity**							
**Caucasian (REF)**	**3866 (85)**	**1210 (31)**	**2656 (69)**	**1.00**		**1.00**	
**Indigenous/First Nations/Metis/Inuit**	**228 (5)**	**63 (28)**	**165 (72)**	**0.84**	**0.62–1.13**	**0.84**	**0.61–1.17**
**Chinese/Filipino/Other Asian**	**124 (3)**	**34 (27)**	**90 (73)**	**0.76**	**0.44–1.30**	**0.82**	**0.53–1.27**
**Caribbean/South American/African**	**70 (2)**	**18 (26)**	**52 (74)**	**2.20**	**0.91–5.29**	**0.54**	**0.30–0.96**
**Middle Eastern/Central Asian/South Asian**	**69 (2)**	**32 (46)**	**37 (54)**	**1.89**	**1.18–3.06**	**1.42**	**0.84–2.40**
**Other**	**146 (3)**	**37 (25)**	**109 (75)**	**0.75**	**0.51–1.09**	**0.85**	**0.56–1.28**
Political Leaning							
Very Liberal	603 (13)	283(47)	320 (53)	2.56	2.07–3.17	2.38	1.89–3.00
Liberal	805 (18)	377 (47)	428 (53)	2.55	2.10–3.11	2.29	1.86–2.83
Slightly liberal	433 (10)	184 (43)	249 (58)	2.14	1.70–2.71	1.91	1.49–2.45
Moderate/middle of the road (REF)	1,029(23)	264 (26)	765 (74)	1.00		1.00	
Slightly Conservative	485 (11)	117 (24)	368 (76)	0.92	0.72–1.18	0.92	0.71–1.19
Conservative	807 (18)	139 (17)	668 (83)	0.60	0.48–0.76	0.63	0.49–0.80
Very Conservative	336 (7)	29 (9)	307 (91)	0.27	0.18–0.41	0.32	0.21–0.48

*OR = Odds ratios are the odds of downloading a contact tracing app compared with the odds of not downloading

**‡**Adjusted OR = Odds ratio adjusted for sex, age, province of residence, household income, highest level of education, ethnicity and political leaning.

^^^ABTraceTogether was the only app available in Alberta at the time of survey

^^^^COVID Alert was available but not widely promoted in British Columbia at the time of survey

^+^Prairie provinces included Saskatchewan and Manitoba; Atlantic provinces include Nova Scotia, New Brunswick, Prince Edward Island and Newfoundland and Labrador

### Factors associated with downloading an app

App downloads differed by sex, province of residence, household income, highest level of education, and political leaning (Table 1). Male participants had lower odds of downloading an app (aOR 0.86, 95%CI 0.75–0.99) compared with females. When compared with the reference category for household income (<$50,000 CAD), the adjusted odds of downloading an app were 2.06 times (95%CI 1.44–2.95) higher for the highest level of household income (≥$200,000). For education, the adjusted odds of downloading an app were 1.84 (95%CI 1.47–2.29) greater for the highest level (university degree) than the reference level (high school graduate or less). In terms of geographic location, individuals living outside of Alberta and British Columbia had higher odds of app download. Persons’ reporting to be liberal had higher odds of downloading an app (aOR 2.29, 95%CI 1.86–2.83), whereas persons reporting to be conservative had lower odds (aOR 0.63 95%CI 0.49–0.80) compared to persons reporting to be moderate/middle of the road in political leaning.

### Reasons for not downloading an app

**Fig 1:**
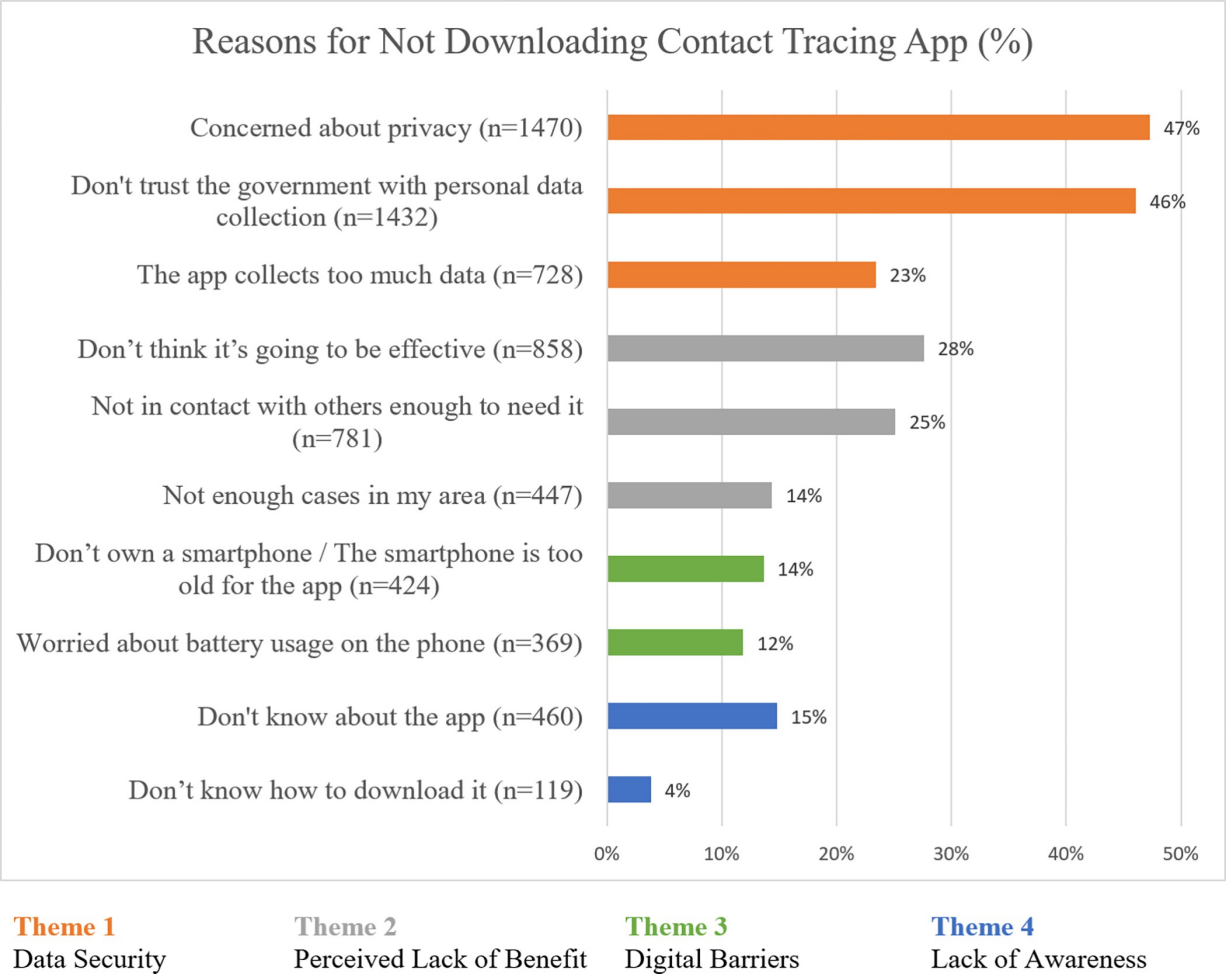
Reasons selected as contributing factors in the decision to not download a contact tracing app (n = 3109). Participants could choose more than one reason.

For the 3,109 participants who had not downloaded an app, the two most often cited reasons were concerns about privacy (n = 1470; 47%) and lack of trust in the government with personal data collection (n = 1432; 46%; Fig 1). Of note, 460 (15%) of those who did not download reported that they were not aware of the app.

### Factors associated with not downloading an app

Compared with female participants, male participants had lower odds of citing reasons related to privacy concerns (OR 0.86, 95%CI 0.75–0.99; Table 2), trust in government (OR 0.62, 95%CI 0.53–0.71), and perceived app ineffectiveness (OR 0.66, 95%CI 0.57–0.78) as contributing to their decision for not downloading an app.

**Table 2:** Associations between demographic characteristics and reasons for not downloading an app. Participants could choose more than one reason.

	Odds for the reasons reported that participants have not downloaded an app									
Characteristic	Theme 1 Data Security			Theme 2 Perceived Lack of Benefit			Theme 3 Digital Barriers		Theme 4 Lack of Awareness	
OR* (95% CI)	Concerned about privacy (n = 1470)	Don’t trust the government with personal data collection (n = 1432)	The app collects too much data (n = 728)	Don’t think it’s going to be effective (n = 858)	Not in contact with others enough to need (n = 781)	Not enough cases in my area (n = 447)	Don’t own a smartphone/ smartphone is too old (n = 424)	Worried about battery usage on the phone (n = 369)	Don’t know about the app (n = 460)	Don’t know how to download it (n = 119)
Biologic Sex										
Female (REF)	1.00	1.00	1.00	1.00	1.00	1.00	1.00	1.00	1.00	1.00
Male	**0.86(0.75–0.99)**	**0.62(0.53–0.71)**	**0.83(0.70–0.98)**	**0.66(0.57–0.78)**	1.19(1.01–1.40)	0.89(0.73–1.08)	**1.40(1.14–1.72)**	1.14(0.92–1.42)	1.90(0.90–1.34)	1.40(0.97–2.03)
Age (years),										
18–34 (REF)	1.00	1.00	1.00	1.00	1.00	1.00	1.00	1.00	1.00	1.00
35–54	1.12(0.94–1.34)	**1.29(1.08–1.55)**	0.89(0.73–1.00)	1.14(0.93–1.38)	0.83(0.68–1.02)	0.84(0.65–1.07)	**1.70(1.13–2.54)**	**0.65(0.51–0.82)**	**0.41(0.32–0.52)**	1.28(0.62–2.64)
55+	**0.50(0.42–0.60)**	**0.65(0.54–0.77)**	**0.42(0.34–0.52)**	**0.77(0.63–0.94)**	0.97(0.79–1.18)	0.81(0.63–1.04)	**8.42(5.91–11.99)**	**0.22(0.16–0.30)**	**0.43(0.34–0.55)**	**5.97(3.24–10.98)**
Province										
AB (REF)	1.00	1.00	1.00	1.00	1.00	1.00	1.00	1.00	1.00	1.00
BC	**0.50(0.40–0.63)**	**0.35(0.27–0.44)**	**0.58(0.44–0.76)**	**0.47(0.35–0.62)**	1.19(0.87–1.62)	1.19(0.87–1.62)	**2.14(1.56–2.94)**	**0.51(0.35–0.76)**	**2.08(1.61–2.68)**	**3.00(1.93–4.65)**
Prairie^+^	0.98(0.76–1.26)	0.81(0.63–1.04)	0.89(0.67–1.19)	1.09(0.83–1.43)	1.29(0.91–1.83)	1.30(0.91–1.83)	**1.57(1.06–2.32)**	0.67(0.44–1.01)	0.71(0.49–1.84)	1.29(0.68–2.46)
ON	**0.72(0.58–0.90)**	**0.54(0.43–0.68)**	**0.69(0.53–0.89)**	0.02(0.72–1.17)	1.28(0.95–1.74)	1.28(0.95–1.74)	**2.58(1.90–3.49)**	0.76(0.54–1.06)	**0.33(0.21–0.50)**	0.73(0.37–1.46)
QC	**0.56(0.44–0.73)**	**0.43(0.33–0.56)**	**0.69(0.53–0.89)**	1.22(0.94–1.59)	0.28(0.15–0.51)	0.28(0.15–0.51)	**3.55(2.58–4.88)**	**0.51(0.33–0.80)**	**0.25(0.14–0.43)**	0.28(0.20–1.26)
Atlantic^+^	**0.50(0.36–0.70)**	**0.35(0.25–0.50)**	**0.50(0.33–0.78)**	**0.61(0.41–0.91)**	**4.16(2.94–5.91)**	**4.16(2.94–5.91)**	**3.32(2.22–4.98)**	**0.47(0.26–0.86)**	0.52(0.49–1.04)	0.74(0.27–2.09)

*Odds ratios are the odds of downloading an app compared with the odds of not downloading

^+^Prairie provinces included Saskatchewan and Manitoba; Atlantic provinces include Nova Scotia, New Brunswick, Prince Edward Island and Newfoundland and Labrador

Statistically significant estimates are bolded

Similarly, compared with participants 18–29 years of age, participants ≥55 years had lower odds of citing reasons related to privacy concerns (OR 0.50, 95%CI 0.42–0.60), trust in government (OR 0.65, 95%CI 0.54–0.77), too much data collection (OR 0.42, 95%CI 0.34–0.52), and perceived app ineffectiveness (OR 0.77, 95%CI 0.63–0.94) as contributing to their decision for not downloading an app. This same group was more likely to state not having an appropriate smartphone (OR 8.42, 95%CI 5.91–11.99) and not knowing how to download the app (OR 5.97, 95%CI 3.24–10.98).

In terms of geography, non-prairie provinces were less likely to cite reasons related to privacy concern, trust in government, and too much data collection as contributing to their decisions for not downloading an app. Of the jurisdictions outside of Alberta, British Columbia was the only province where people had higher odds of not knowing about the app (OR 2.08, 95%CI 1.61–2.68) or not knowing how to download the app (OR 3.00, 95%CI 1.93–4.65). Finally, outside of Alberta, participants had higher odds of not owning an appropriate smartphone but lower odds of worrying about phone battery usage by the app.

Participants who did not have COVID Alert outside of Alberta were asked if they would download the app in the future: 110 (7%) selected “Yes”, 690 (44%) answered “No”, and 758 (49%) responded “Maybe” or “Not Sure”. Male participants (aOR 0.75, 95%CI 0.60–0.95) and those who had data security concerns, did not own a smartphone (aOR 0.60 95%CI 0.45–0.81), and perceived limited effectiveness (aOR 0.53, 95%CI 0.41–0.69) were less likely to consider downloading the app (Table 3). Those who perceived limited benefit due to lack of cases (aOR 1.51, 95%CI 1.20–2.08) or lack of contacts (aOR 1.59, 95%CI 1.23–2.05) were more likely to consider downloading an app. Individuals who did not know about the app or how to download it were more likely to consider future uptake.

**Table 3:** Associations between COVID-19 contact tracing applications downloads and trusted sources of COVID-19 information.

	Total N = 4503 (%)	Downloaded a Contact Tracing App		cOR	95% CI	aOR	95% CI
		Yes (N = 1394)	No (N = 3109)				
**Most Trusted Sources for COVID-19 Information** *							
Chief Medical Officer of Health	1935 (43)	773 (40)	1162 (60)	1.89	1.66–2.12	1.83	1.59–2.12
Media Briefings (Federal or Provincial)							
Public Health Websites	1758 (39)	731 (42)	1027 (58)	2.04	1.79–2.33	1.57	1.36–1.81
Healthcare Provider	1239 (28)	423 (34)	816 (66)	1.13	0.98–1.30	1.12	0.96–1.30
Television/Radio News	607 (13)	172 (28)	435 (72)	0.81	0.66–0.97	0.84	0.68–1.03
Internet Searches (e.g., Google)	529 (12)	72 (14)	457 (86)	0.29	0.22–0.38	0.38	0.29–0.50
Friends and Family	159 (4)	22 (14)	137 (86)	0.33	0.21–0.51	0.50	0.31–0.80
Print Newspaper	134 (3)	41 (31)	93 (69)	0.92	0.64–1.34	0.86	0.57–1.29
**Most Trusted Social Media Platforms for COVID-19 Information** *							
Facebook	2169 (48)	637 (34)	1532 (71)	0.82	0.72–0.93	0.91	0.78–1.05
YouTube	978 (22)	255 (26)	723 (74)	0.71	0.61–0.83	0.80	0.68–0.96
Twitter	797 (4)	343 (43)	454 (57)	1.86	1.58–2.17	1.67	1.40–1.99
Instagram	452 (10)	167 (37)	285 (63)	1.31	1.06–1.60	1.07	0.86–1.34
Reddit	407 (9)	187 (46)	220 (54)	1.98	1.61–2.43	1.48	1.17–1.86
TikTok	53 (1)	24 (45)	29 (55)	1.81	1.05–3.12	1.72	0.95–3.11
Snapchat	39 (1)	7 (18)	32 (82)	0.47	0.21–1.07	0.73	0.30–1.76
Twitch	8 (1)	1 (13)	7 (88)	0.31	0.04–2.52	0.34	0.04–2.86
Dating Apps (e.g., Tinder)	6 (1)	1 (17)	5 (83)	0.43	0.05–3.71	0.67	0.07–6.08
Other	168 (4)	46 (27)	122 (76)	0.81	0.57–1.15	0.90	0.62–1.31
Does Not Use Social Media	205 (5)	38 (19)	167 (81)	0.49	0.35–0.71	0.68	0.47–1.00

*Participants could pick more than one most trusted source from each list

cOR = crude odds ratio

aOR = adjusted odds ratio, adjusted for sex, province of residence, highest education, political leaning and income level

### Associations between downloading an app and trusted sources of COVID-19 information

Persons who trusted COVID-19 information from a Chief Medical Officer of Health (aOR 1.83, 95%CI 1.59–2.12) and public health websites (aOR 1.57, 95%CI 1.36–1.81) had higher odds of downloading an app than those who did not trust these sources. Those who reported their most trusted COVID-19 information were internet searches (aOR 0.38, 95%CI 0.29–0.50) or friends and family (aOR 0.50, 95%CI 0.31–0.80) had lower odds of app download than those who did not trust these sources (Table 4).

**Table 4:** Associations between demographic characteristics, reasons for not downloading an app, and potential for downloading a contact tracing app in the future.

Characteristic	Might Download App in the Future*			
	cOR*	95% CI	aOR	95% CI
Biologic Sex, N (%)				
Female (REF)	1.00		1.00	
Male	0.67	0.55–0.83	0.75	0.60–0.95
Age (years)				
18–34 (REF)	1.00		1.00	
35–54	0.76	0.58–1.00	0.87	0.64–1.17
55+	1.02	0.79–1.33	1.29	0.97–1.72
Reasons for not downloading an app^+^				
Concerned about privacy	0.57	0.47–0.71	0.62	0.49–0.78
Did not trust the government with personal data collection	0.25	0.20–0.32	0.30	0.24–0.39
The app collects too much data	0.30	0.23–0.39	0.31	0.23–0.42
Don’t think it’s going to be effective	0.42	0.33–0.53	0.53	0.41–0.69
Not in contact with others enough to need it	1.58	1.24–2.01	1.59	1.23–2.05
Not enough cases in my area	1.58	1.18–2.1	1.51	1.20–2.08
Don’t own a smartphone/The smartphone is too old for the app	0.75	0.57–0.98	0.60	0.45–0.81
Worried about battery usage on the phone	1.02	0.72–1.44	0.90	0.62–1.30
Did not know about the app	3.90	2.66–5.73	3.01	1.99–4.56
Don’t know how to download it	3.90	2.66–5.73	3.69	1.64–8.29

*For those that did not have COVID Alert in the provinces outside of AB, they were asked if they would download it in the future. Odds ratios are the odds of might download (“Maybe” n = 550 or “Not Sure” n = 208) compared with the odds of will not download (“No” n = 690) the app in the future.

^+^Participants could choose more than one reason.

cOR = crude odds ratio

aOR = adjusted odds ratio, adjusted for sex, province of residence, highest education, political leaning and income level

Persons who reported their most trusted social media platforms for COVID-19 information were YouTube (aOR 0.80, 95%CI 0.68–0.96) had lower odds of app download than those who did not. Persons who reported their most trusted social media platforms for COVID-19 information were Twitter (aOR 1.67, 95%CI 1.40–1.99) and Reddit (aOR 1.48, 95%CI 1.17–1.86) had greater odds of downloading an app than those who did not trust those platforms.

## Discussion

This cross-sectional study analyzed demographic and socioeconomic factors associated with downloading and reasons for not downloading contact tracing and exposure notification apps. Demographic and socioeconomic factors positively associated with downloading an app were: 1) being female, 2) higher household income, 3) higher education level attained, and 4) more liberal political views. The most prominent theme for not downloading an app was related to data security, which was more influential for female and younger participants. The second most common theme was perceived lack of benefit. Demographic associations were less consistent with this theme, though older female participants were more likely to believe apps were ineffective and male participants were more likely to believe that they did not need an app. The third most common theme centered on digital barriers, which were more likely to impact downloading by older participants. Health-related information sources and certain social media platforms (e.g., Twitter, Reddit) were also positively associated with downloading an app. In contrast, non-health information sources (e.g., television, internet search), Facebook, and YouTube were negatively associated with app download.

Our research represents the first large-scale Canadian study investigating factors associated with app download and adds to the growing evidence base to help inform better design, usability, and promotion of these apps. The associations between higher income and higher education with app download are consistent with previous studies on public attitudes towards apps, though unlike previous studies, our model did not show associations between age and downloading an app [39–43]. We found that downloading an app varied by sex, which has not been reported in other studies [40, 44, 45]. Moreover, the identified differential app download rate along the political spectrum is in direct contrast to surveys of American perception which found relatively small difference in support for apps between Democrats and Republicans [19]. The most prominent concerns associated with not downloading an app (i.e., privacy, trust in government, data security) are consistent with literature evaluating public perception of apps prior to their wide implementation [16, 17, 39, 44]. Survey studies after the wide implementation of apps have reported similar results [21, 22, 46]. We found a lower likelihood of app download in Alberta and British Columbia, which may be due to lack of public promotion as shown by the fact that residents of these two provinces were more likely to cite not knowing about the apps as a reason for not downloading an app.

Our findings can guide app promotion to increase app downloads. First, app distributors can more efficiently use promotional resources by targeting specific demographics (i.e., male, lower household income, lower education level, conservative political leaning) with low download patterns. Second, governments can use specific tactics to address concerns voiced by non-adopters. Privacy is the biggest concern and studies show that people are most willing to accept apps from health protection agencies [41]. However, both the ABTraceTogether and COVID Alert webpages are hosted by centralized government websites, and could instead be hosted by decentralized public health agencies. Research has also shown that people value decentralized app data storage [19], which is the form that both Canadian apps use; this fact could be better highlighted or explained on app websites to assuage fears around data security. Finally, health-related information sources should better utilize and target specific social media platforms such as Facebook and YouTube for app promotion.

Future studies should examine additional measures in the Canadian setting such as: app quality using a validated tool such as the Mobile App Rating Scale; user engagement with the app after downloading; positive predictive value, sensitivity, and contact yield of the apps; user compliance with self-isolation and public health recommendations after receiving exposure notification; and real-world effectiveness based on averted public exposure and outbreak events [47–49]. These factors will be critical in slowing spread of infection as new COVID-19 variants of concern emerge.

## Limitations

There are several limitations to our work. This was a cross-sectional survey representing persons’ attitudes and behaviors at the time of this study, which will continue to change over time as the pandemic evolves. The survey recruited participants from an existing voluntary national panel designed to be representative of the Canadian population, but is not immune to selection bias as demonstrated by the proportion of respondents who had downloaded an app being greater than the estimated uptake across Canada. This discrepancy is likely due to panel volunteers having greater social responsibility and technological literacy, and therefore being more likely to download an app. In addition, the Hawthorne effect may have limited contribution towards participants falsely endorsing downloading an app. Furthermore, the study population had a greater proportion of participants with post-secondary education and identifying as Caucasian than the general population of Canada. A non-response bias is possible as 1,388 participants began but never completed the survey. Despite this being a large national survey, we were constrained by a small sample size for certain analyses. Finally, this study uses downloading of an app as a proxy for app adoption, which may not hold true since individuals can download the app but never use it and we did not capture whether individuals used the app after downloading it in our survey.

## Conclusions

Our findings can inform design and promotion of contact tracing and exposure notification apps to increase adoption and slow transmission of COVID-19. Targeted messaging can be designed and directed to population segments less likely to download an app (e.g., males) using information platforms associated with lower app downloads (e.g., Facebook, YouTube). Moreover, promotional resources and updates to the apps need to focus on addressing public concerns around data security and perceived lack of benefit from using these apps, which can be done through persuasive design to make the apps more appealing and motivational [25]. Finally, better communication regarding data security features, such as decentralized data storage, and hosting these apps on public health agency websites may increase public trust and adoption.
